# Development and functional validation of a disulfidoptosis-related gene prognostic model for lung adenocarcinoma based on bioinformatics and experimental validation

**DOI:** 10.3389/fimmu.2025.1540578

**Published:** 2025-02-10

**Authors:** Tao Shen, Zhuming Lu, Sisi Yang, Dongxi Zhang, Yongwen Ke, Zhuowen Chen, Jinqiang Wu, Weidong Wu

**Affiliations:** ^1^ Department of Surgery, First Clinical Medical College of Jinan University, Guangzhou, Guangdong, China; ^2^ Department of Thoracic Surgery, Jiangmen Central Hospital, Jiangmen, Guangdong, China; ^3^ Department of Psychology, Jiangmen Third People’s Hospital, Jiangmen, Guangdong, China; ^4^ Department of Cardiothoracic Vascular Surgery, The Affiliated Hospital of Youjiang Medical University for Nationalities, Baise, Guangxi, China; ^5^ Baise Key Laboratory of Molecular Pathology in Tumors, Baise, Guangxi, China; ^6^ Department of Cardiothoracic Vascular Surgery, Guangzhou Red Cross Hospital of Jinan University, Guangzhou, Guangdong, China

**Keywords:** LUAD, disulfidptosis, tumor microenvironments, prognosis, immunotherapy

## Abstract

**Background:**

Disulfidoptosis is increasingly linked to cancer progression, yet its immunological impacts and prognostic value in lung adenocarcinoma (LUAD) remain poorly understood. This study aims to delineate the predictive significance of disulfidoptosis-related genes (DRGs) in LUAD, their potential as therapeutic targets, and their interaction with the tumor microenvironment.

**Methods:**

We analyzed the expression profiles of 23 DRGs and survival data, performing consensus clustering to identify molecular subtypes. Survival analysis and gene set variation analysis (GSVA) were used to explore cluster differences. Key DRGs were selected for Cox and LASSO regression to develop a prognostic model. Tensin4 (TNS4), a key gene in the model, was further evaluated through immunohistochemistry (IHC) in LUAD and normal tissues and gene knockdown experiments *in vitro*.

**Results:**

Two clusters were identified, with 225 differentially expressed genes. A six-gene signature was developed, which classified LUAD patients into high- and low-risk groups, showing significant survival differences. The risk score independently predicted LUAD prognosis and correlated with immunotherapy responses. IHC showed elevated TNS4 levels in LUAD tissues, while *in vitro* TNS4 knockdown reduced both cell proliferation and migration.

**Conclusion:**

This study highlights the role of DRGs in LUAD, with a validated gene signature offering new avenues for targeted therapies, potentially improving LUAD treatment outcomes.

## 1 Introduction

Lung cancer remains the primary cause of cancer-related mortality worldwide, with an estimated 1.8 million deaths annually (1, 2). In China, it tops the list of cancer incidences with around 1,060,600 new cases reported in 2022, making up 22.0% of the total cancer diagnoses (3). Lung adenocarcinoma (LUAD), the most common form of non-small cell lung cancer (NSCLC), represents about 40% of all lung cancer diagnoses (4). Despite available treatments such as surgery, radiation, chemotherapy, immunotherapy, and targeted therapy, the five-year survival rate for lung cancer hovers around 19.7% (5, 6). Thus, improving survival rates, enhancing quality of life, and precisely predicting tumor prognosis are critical challenges in cancer treatment (7).

Disulfidoptosis represents a unique type of cellular demise that deviates from conventional pathways of programmed cell death, primarily due to a disruption in cysteine uptake and NADPH availability. This deficiency in NADPH leads to the formation of abnormal disulfide bonds in actin cytoskeletal proteins. Such anomalies result in an accumulation of disulfide compounds, inducing disulfide stress that adversely affects the actin cytoskeleton. The resulting sequential disturbances dismantle the actin structure, ultimately leading to cell death (8–10).

In this study, we have pinpointed genes regulated by disulfidptosis that enable molecular subtyping of LUAD. Furthermore, we established a risk score model utilizing disulfidoptosis-related genes (DRGs) to forecast LUAD patient outcomes. The model stratified patients into high-risk and low-risk groups, examining their survival rates, tumor immune environments, and responses to immunotherapy. These findings emphasize the role of disulfidptosis as a strong prognostic indicator and a potential therapeutic target in LUAD.

## 2 Materials and methods

### 2.1 Data collection

We obtained LUAD datasets from the Cancer Genome Atlas (TCGA, https://www.cancer.gov). The selection criteria included histological confirmation of malignant LUAD and the availability of RNA expression profiles alongside overall survival (OS) statistics. The cohort under study included 561 patients.

### 2.2 CNV analysis and DRG cluster establishment

Gene expression differences between tumor and adjacent normal tissues were examined using the “limma” R package (11). Mutations in LUAD samples from TCGA were characterized for frequency using the “maftools” script (12, 13). We analyzed copy number variations (CNVs), which involve changes in the number of copies of genomic segments, using the “RCircos” package (14). The clustering of LUAD patients was performed using “ConsensusCluster Plus,” based on an optimal number of clusters identified at the inflection point of the sum of squared errors (SSE) (15, 16).

### 2.3 Functional enrichment analysis

Functional enrichment analysis was performed on the identified clusters using the “GSVA” and “GSEABase” packages to uncover pathways (17). Differential gene expression was analyzed using the “limma” package, with a significance cutoff of *p* < 0.05. The exploration of gene functions and pathways involved in Gene Ontology (GO) and Kyoto Encyclopedia of Genes and Genomes (KEGG) was facilitated through “ggplot2,” which generated histograms, bubble, and circle diagrams.

### 2.4 Construction and validation of the risk-scoring model

Initial screening with a univariate Cox model linked 225 DRGs with patient OS, identifying 114 significant genes (*p* < 0.05). The LASSO technique was applied to prevent overfitting within the TCGA cohort. A multivariate Cox model was used to formulate a prognostic risk-scoring equation based on the expression of specific genes (18, 19). Risk score=[(0.412× Expression value of ZMAT4)+(0.667× Expression value of AL031258.1)+[(-1.198)× Expression value of LINC01374]+[(-1.117)×Expression value of AP002358.1]+(0.167× Expression value of TNS4)+(0.169× Expression value of NAMPTP1)+(1.201× Expression value of SCN5A)+(0.321× Expression value of KLK8)+(0.782× Expression value of NOL4)+(1.919× Expression value of AC104794.5)+(0.542× Expression value of LINC00941)]. Patients were categorized into high- or low-risk categories according to the median risk score. Differences in survival were analyzed using log-rank tests and visualized through Kaplan-Meier plots, along with graphs depicting survival status, OS, and risk score distributions within the training set (*p* < 0.05) (20).

### 2.5 Independent prognostic evaluation

The “Survival” package in R was used to perform both univariate and multivariate Cox regression analyses to assess the impact of clinicopathological factors and risk scores on survival outcomes. Prognostic accuracies were assessed using time-dependent ROC curves via the “timeROC” package, with a significance level set at *p* < 0.05 (21–23).

### 2.6 Subgroup analysis

Our model’s predictive value was further evaluated by dividing patients into specific subgroups according to various factors. These included age groups (≤65 and >65 years), tumor stages (I-II and III-IV), T stages (T1-2 and T3-4), N stages (N0 and N1-3), M stages (M0 and M1), and gender (male and female). Survival predictions were performed for each category, achieving statistical significance with p-values below 0.05.

### 2.7 Tumor microenvironment and immune profiling in LUAD

We applied ESTIMATE and CIBERSORT algorithms to define the tumor microenvironment score and delineate the composition of 22 immune cell subsets (24–26). Additionally, we examined variations in immune checkpoint expressions across risk groups, noting significance at *p* < 0.05.

### 2.8 Tumor mutation burden assessment

TMB was calculated using somatic mutation data from the TCGA LUAD dataset, analyzed with “maftools” (27). We visualized TMB distribution via a waterfall chart and examined correlations between TMB scores and risk scores with survival outcomes, categorizing patients into high- and low- TMB groups for Kaplan-Meier analysis with “Survival” and “survminer” packages (28).

### 2.9 Bioinformatics insights into TNS4 as a prognostic indicator

First, we evaluated the expression levels of Tensin4 (TNS4) in LUAD compared to normal lung tissues, as depicted in box plots. The Kruskal-Wallis Rank Sum Test was utilized to analyze the expression levels of TNS4 across different stages in LUAD. Survival impacts of TNS4 expression were assessed using Kaplan-Meier survival curves. Lollipop charts illustrated the infiltration of immune cells related to TNS4, examining their associations through Spearman correlation analysis Additionally, the “clusterProfiler” package facilitated GSEA to elucidate the biological activities associated with TNS4 in the prognosis of LUAD (29, 30).

### 2.10 Immunohistochemical analysis of TNS4 in LUAD

Immunohistochemical testing was conducted on 3 µm sections derived from formalin-fixed, paraffin-embedded samples to assess TNS4 expression levels in LUAD relative to lung tissues. The study included 90 LUAD cases and 11 controls. Sections were dried at 67°C for three hours, dewaxed in xylene, rehydrated in graded alcohols, and subjected to antigen retrieval using EDTA. Incubation with TNS4 polyclonal antibodies (Proteintech, 1:150) occurred overnight at 4°C. Detection involved biotin-conjugated secondary antibodies and horseradish peroxidase complexes with diaminobenzidine. Staining intensity was quantified by three independent pathologists through integrated optical density across five fields. The study received ethical approval from Jiangmen Central Hospital’s Clinical Research Ethics Committee (Approval No. 2024–238A).

### 2.11 Cell culture

The LUAD cell lines A549 and H1299 were cultured in RPMI 1640 medium enriched with 10% fetal bovine serum, within a 5% CO2 environment at 37°C. These cell lines were sourced from the American Type Culture Collection (ATCC).

### 2.12 RNA interference and transfection

Specific siRNAs targeting TNS4 were procured from Shanghai GenePharma Co. Ltd. A549 and H1299 cells underwent transfection with 50 nmol/L siRNA using Lipofectamine 2000. Knockdown efficiency was verified through RT-qPCR. SiRNA sequences included: si-TNS4-1: 5’-GCAUCUCAAUCCCUUGCAUTT-3’, si-TNS4-2: 5’-CCAAAGGAGUGCAUCUCAATT-3’.

### 2.13 RT-qPCR

Total RNA was extracted using Trizol reagent (Vazyme), and converted to cDNA using HiScript III RT SuperMix (Vazyme). The RT-qPCR analyses were carried out with Universal SYBR Green Fast qPCR Mix, with expressions quantified via the 2^(-ΔΔCt)^ method (31–33). GAPDH served as the reference gene. Primer sequences were: GAPDH, F-5’-GGCTGTTGTCATACTTCTCATGG-3’, R-5’- GGAGCGAGATCCCTCCAAAAT-3’. TNS4, F-5’- TGTTTGGAAGCAATCAGTCCCT-3’, R-5’- TACTAGGAGCCTGGGCATCA -3’.

### 2.14 Clone formation tests

Post-transfection, cells were plated in 6-well plates to evaluate colony formation over a two-week period. Colonies were then fixed with 4% paraformaldehyde, stained with 0.1% crystal violet, and documented using high-definition photography (34). Analysis was conducted using ImageJ software.

### 2.15 Edu assay

Transfected cells were plated in 24-well plates and incubated with EdU for two hours before fixation with 4% paraformaldehyde. Nuclei staining was conducted using DAPI. EdU incorporation was visualized and quantified using a Nikon microscope and ImageJ software.

### 2.16 Wound healing assay

A scratch was introduced to confluent cell monolayers in 6-well plates using a sterile pipette tip. Post-scratch, the cells were cultured in serum-free medium to inhibit cell proliferation, facilitating the observation of cell migration over 24 hours with an electron microscope (35).

### 2.17 Cell migration assay

The assessment of cell migration was conducted using a transwell setup (Corning, 8 µm pore size). LUAD cells were placed in the upper chamber in serum-free medium, while the lower chamber contained medium enriched with 10% FBS. After 24 hours, cells that migrated through the membrane were fixed, stained with crystal violet, and imaged.

## 3 Result

### 3.1 Genetic variation and expression of DRGs in LUAD

**Figure 1:**
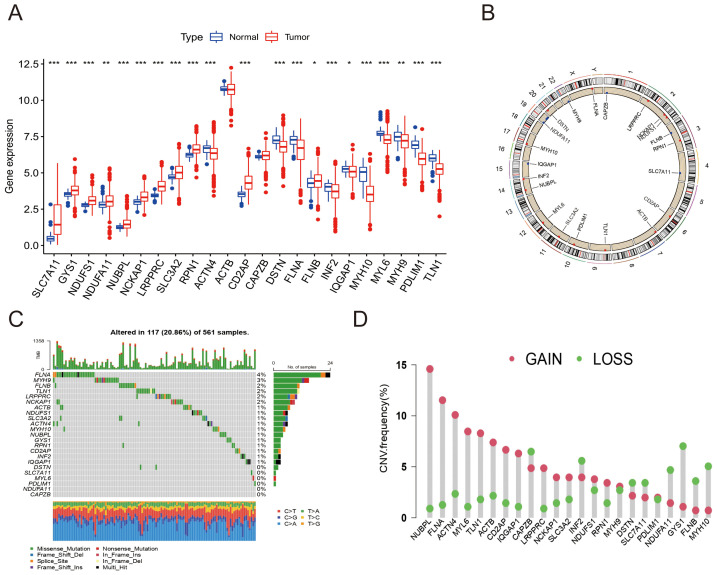
Expression and genomic variations of Disulfidoptosis-Related Genes (DRGs) in lung adenocarcinoma (LUAD). **(A)** Comparative expression of 23 DRGs between LUAD and normal lung tissues (^*^ *p*<0.05; ^**^ *p*<0.01; ^***^ *p*<0.001). **(B)** Locations of copy number variations (CNVs) in DRGs across 23 chromosomes. **(C)** Mutation frequencies of DRGs in 561 LUAD patients. **(D)** Prevalence of CNV gains among DRGs.

We evaluated genetic differences among 23 DRGs between LUAD and normal lung tissues (**Figure 1A**). Significant differences were noted in all but ACTB and CAPZB (*p* < 0.05). Chromosomal locations and copy number variations (CNVs) of these DRGs are depicted in **Figure 1B**. TCGA LUAD data revealed mutations in DRGs across 117 of 561 samples, with FLNA and MYH9 exhibiting the highest mutation frequencies. No mutations were observed in NDUFA11 and CAPZB (**Figure 1C**). CNV analysis showed a predominance of CNV gains (**Figure 1D**).

### 3.2 Clustering and functional analysis of DRGs in LUAD

**Figure 2:**
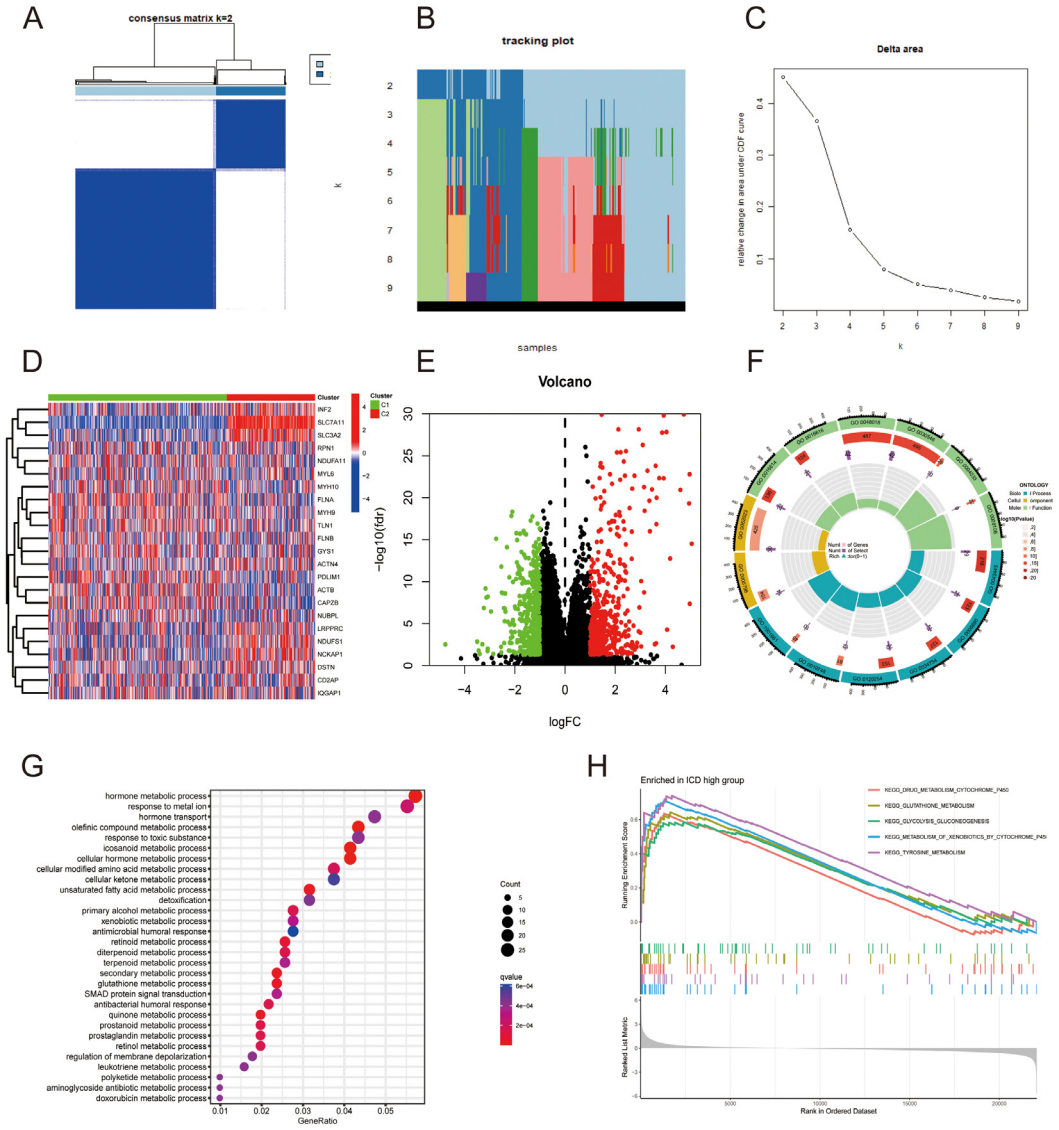
Characterization of Disulfidoptosis-Related Gene (DRG) Subtypes. **(A)** Heatmap from the consensus clustering matrix for two clusters (k = 2). **(B)** Tracking plot of cluster membership. **(C)** Changes in area under the cumulative distribution function (CDF) curve. **(D)** Heatmap visualizing expression level differences between the two clusters. **(E)** Volcano plot displaying up-regulated and down-regulated genes between clusters (t-test, *p* < 0.05). **(F)** Gene Ontology (GO) functional enrichment analysis for the clusters. **(G)** Kyoto Encyclopedia of Genes and Genomes (KEGG) pathway enrichment analysis. **(H)** Gene Set Enrichment Analysis (GSEA) of the clusters.

Consistent clustering of the 23 DRGs identified two main clusters with distinct expression profiles. The optimal cluster stability and internal consistency were achieved with k = 2, as illustrated in **Figures 2A–C**. Expression differences between the clusters were particularly notable between the SLC7A11 and SLC3A2 groups (**Figure 2D**). Volcano plots further delineated these disparities, and we performed GO and KEGG enrichment analyses on the differentially expressed genes (**Figures 2E**). Biological process enrichment included metabolic pathways like quinone, secondary metabolites, and hormone-related processes. DRGs were predominantly associated with the Golgi lumen and extracellular matrix in cellular components, and their molecular functions included activities of receptor ligands and various oxidoreductases (**Figure 2F**). The most enriched pathways identified through KEGG analysis involved hormone metabolic process, response to metal ion, and hormone transport (**Figure 2G**). GSEA indicated significant pathway activations in drug metabolism, glutathione metabolism, and glycolysis (**Figure 2H**). These findings collectively underscore the pivotal role of DRGs in orchestrating diverse biological pathways and cellular processes in LUAD, potentially influencing tumor metabolism and the tumor microenvironment.

### 3.3 Development and validation of a DRG-based risk signature for LUAD

**Figure 3:**
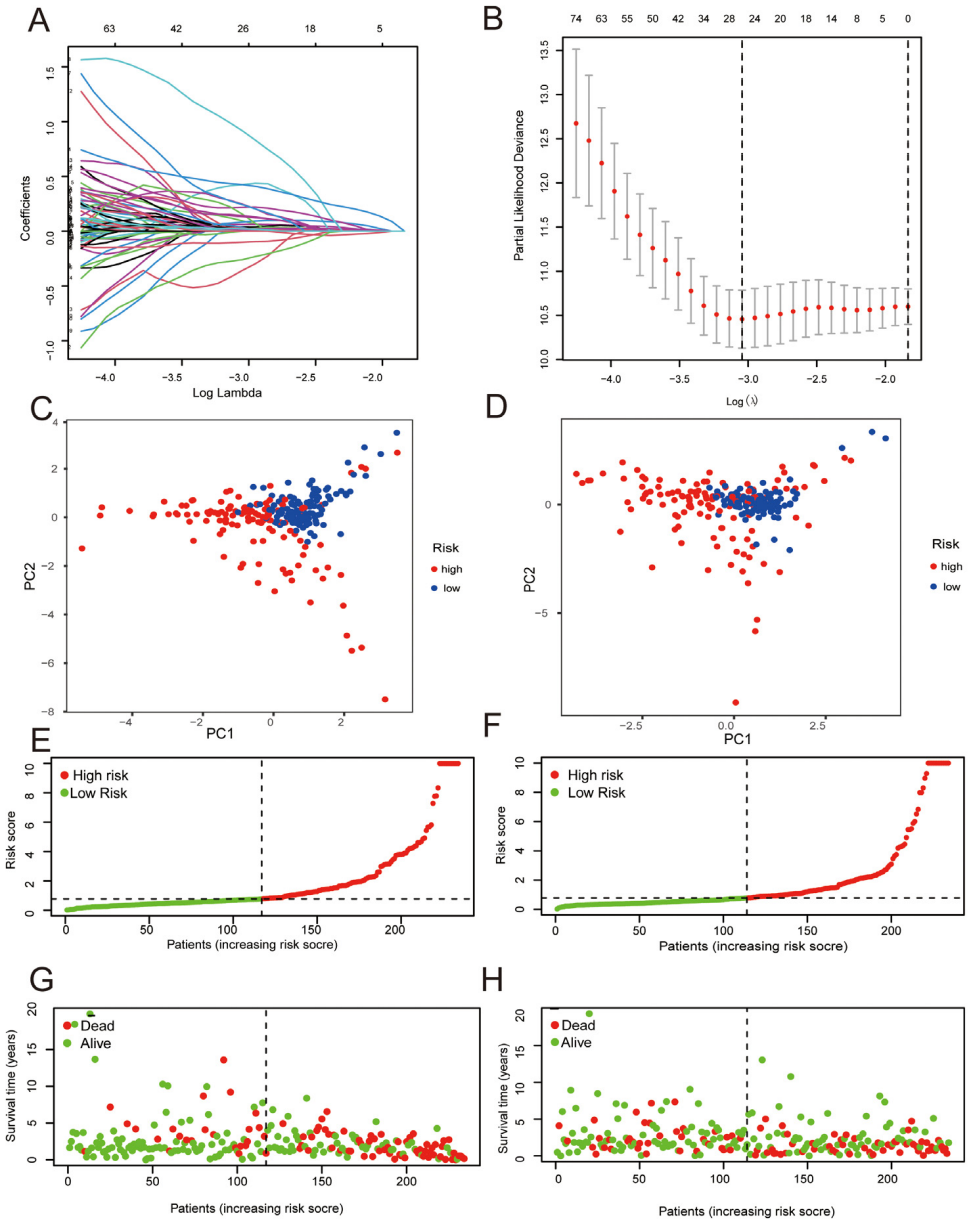
Development of the Lung Adenocarcinoma (LUAD) risk score model. **(A, B)** LASSO coefficient profiles **(A)** and variable selection trajectories during 1,000-fold cross-validation **(B)**. **(C, D)** Principal Component Analysis (PCA) plots showing separation of high- and low-risk groups based on 11 genes within TCGA training and validation cohorts. **(E, F)** Risk score distributions in the training **(E)** and validation **(F)** cohorts. **(G, H)** Survival status and overall survival in relation to risk scores for the training **(G)** and validation **(H)** cohorts.

**Figure 4:**
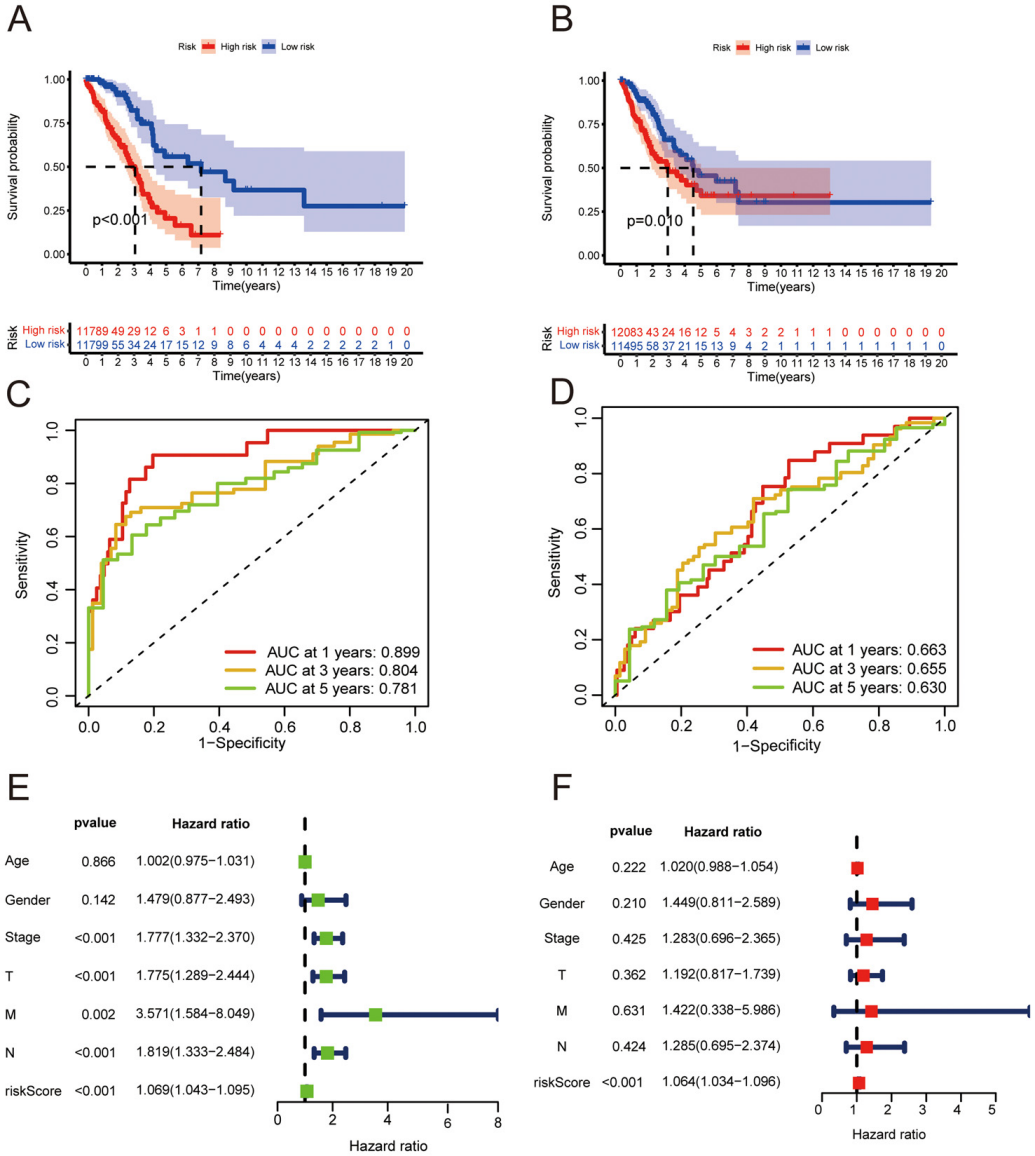
Prognostic validation of the risk score and clinical features. **(A, B)** Kaplan-Meier survival curves for high- and low-risk groups in the training **(A)** and validation sets **(B)** (log-rank test, *p* < 0.05). **(C, D)** ROC curves for 1, 3, and 5-year overall survival based on risk scores in the TCGA training set **(C)** and validation cohort **(D)**. **(E)** Univariate Cox regression analysis of risk factors in lung adenocarcinoma (LUAD) patients. **(F)** Multivariate Cox regression analysis of these factors in LUAD patients.

We identified 967 genes displaying differential expression between two clusters using stringent criteria (|log2FC| > 1 and FDR < 0.05). From these, 159 genes linked to prognosis via univariate Cox regression (*p* < 0.05) were further refined to an 11-gene signature through Lasso-Cox analysis (**Figure 3A**). This signature calculated risk scores for each patient, stratifying them into high and low-risk categories based on median scores. Principal Component Analysis (PCA) revealed distinct genetic profiles between these risk groups, effectively categorizing LUAD patients into separate cohorts (**Figures 3C, D**). Kaplan-Meier survival analysis confirmed a significantly shorter OS for the high-risk group across both training (*p* < 0.001, **Figure 4A**) and validation cohorts (*p* < 0.05, **Figures 4B**). The risk curves and scatter plots illustrated the direct correlation between increased risk scores and mortality rates in these cohorts (**Figures 3E−H**). ROC curve analysis underscored the model’s accuracy with AUC values of 0.899, 0.804, and 0.781 over 1, 3, and 5 years respectively in the training set, and 0.663, 0.655, and 0.630 in the validation set (**Figures 4C, D**). These analyses substantiate the signature as a robust prognostic tool, capable of distinguishing between risk levels and predicting overall survival with high accuracy in patients with LUAD.

### 3.4 Independent prognostic significance of the risk signature

The risk score was confirmed as an independent prognostic factor through univariate and multivariate Cox regression analyses, with hazard ratios (HR) of 1.069 (95% CI: 1.043–1.095, *p* < 0.001) and 1.064 (95% CI: 1.034–1.096, *p <* 0.001) respectively (**Figures 4E, F**). These results affirm the risk score’s prognostic significance, independent of various clinicopathological parameters including M stage, N stage, T stage, tumor stage, age, and gender.

**Figure 5:**
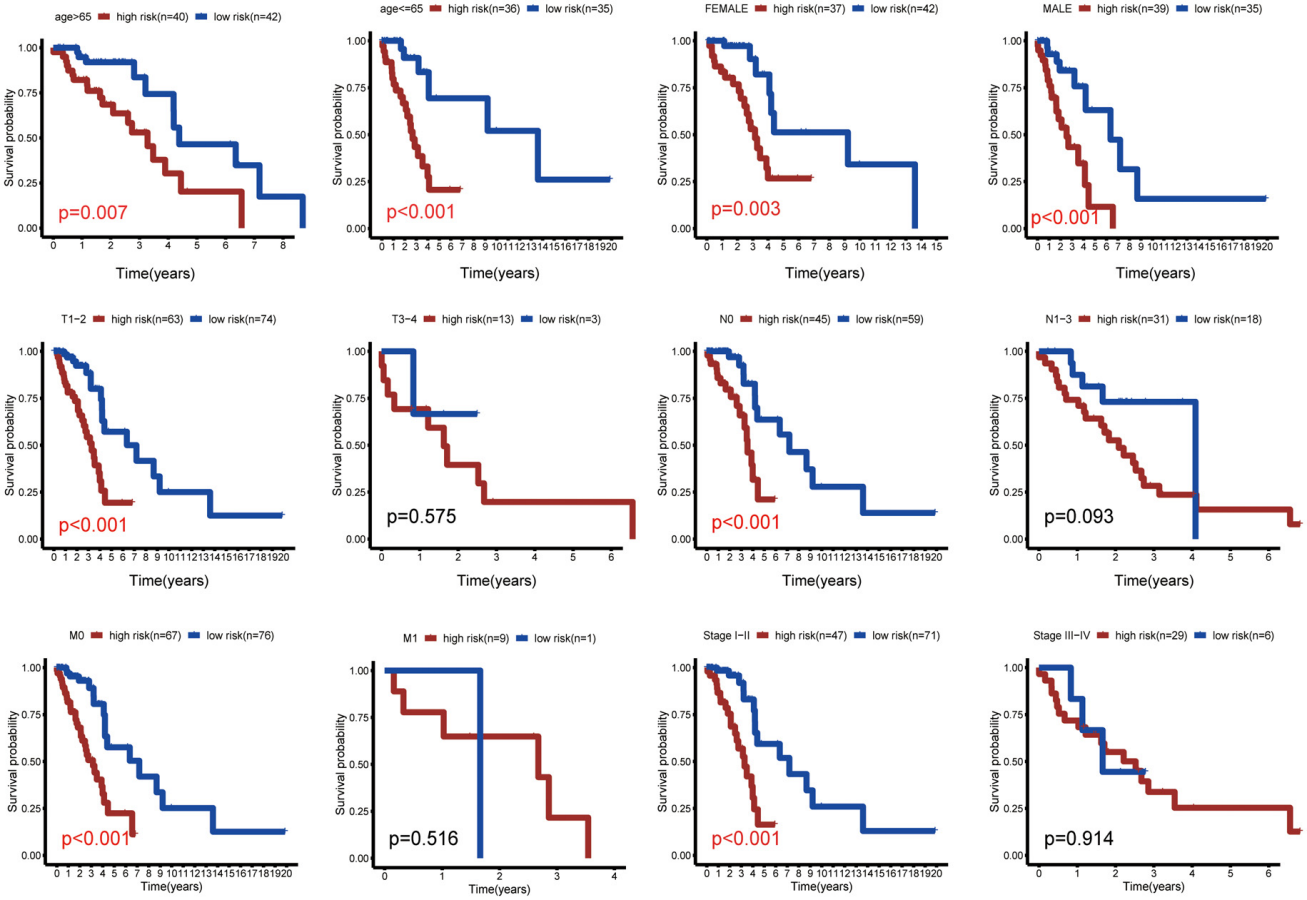
Subgroup analysis of survival outcomes. Kaplan-Meier survival curves comparing high- and low-risk patients across various subgroups, including age, gender, TNM stage, and tumor stage. Significance in survival differences was assessed using log-rank tests.

To further corroborate the clinical independence of the risk score, we conducted subgroup analysis. These analyses consistently showed lower OS for the high-risk group compared to the low-risk group across subgroups including age ≤ 65, age >65, male, female, T1-2, N0, M0, stage I-II (**Figure 5**). This underscores the tight correlation between the risk score and the clinical characteristics of LUAD, showcasing its utility as an effective tool for prognostication.

### 3.5 Tumor microenvironment and immune profiling

CIBERSORT analysis identified significant variations in six key immune cells—resting NK cells, monocytes, M0 macrophages, resting dendritic cells, resting mast cells, and activated mast cells—between high- and low-risk groups (**Figure 7A**), with all differences being statistically significant (p < 0.05). Additionally, ssGSEA revealed enhanced enrichment scores for immune functions such as APC co-inhibition, APC co-stimulation, checkpoint regulation, HLA activity, para-inflammation, and T cell responses in the low-risk group, indicating stronger immune presence compared to the high-risk group (*p* < 0.05, **Figure 7B**). A focused assessment of HLA related genes revealed a higher expression of 20 HLA related genes in the low-risk group, suggesting potential immune engagement differences between the groups (**Figure 7C**). Discrepancies in immune checkpoint-related molecule expression were also noted, pointing to possible immunotherapeutic targets for LUAD patients (**Figure 7D**) (27). The study further linked survival variations to interactions between disulfidoptosis and the tumor immune microenvironment, with higher immune and ESTIMATE scores observed in the low-risk group (*p* < 0.001; **Figure 7E**). TIDE analysis indicated lower potential for immune escape in the high-risk group, suggesting a higher efficacy of immune checkpoint inhibitors in these patients. Conversely, the low-risk group displayed higher T-cell dysfunction scores (**Figures 8A–C**), indicating different immune profiles and responses to therapy. This comprehensive immune profiling elucidates how the immune landscape in LUAD can influence patient prognosis and therapeutic responsiveness, reinforcing the need for personalized immunotherapy approaches based on risk stratification.

**Figure 6:**
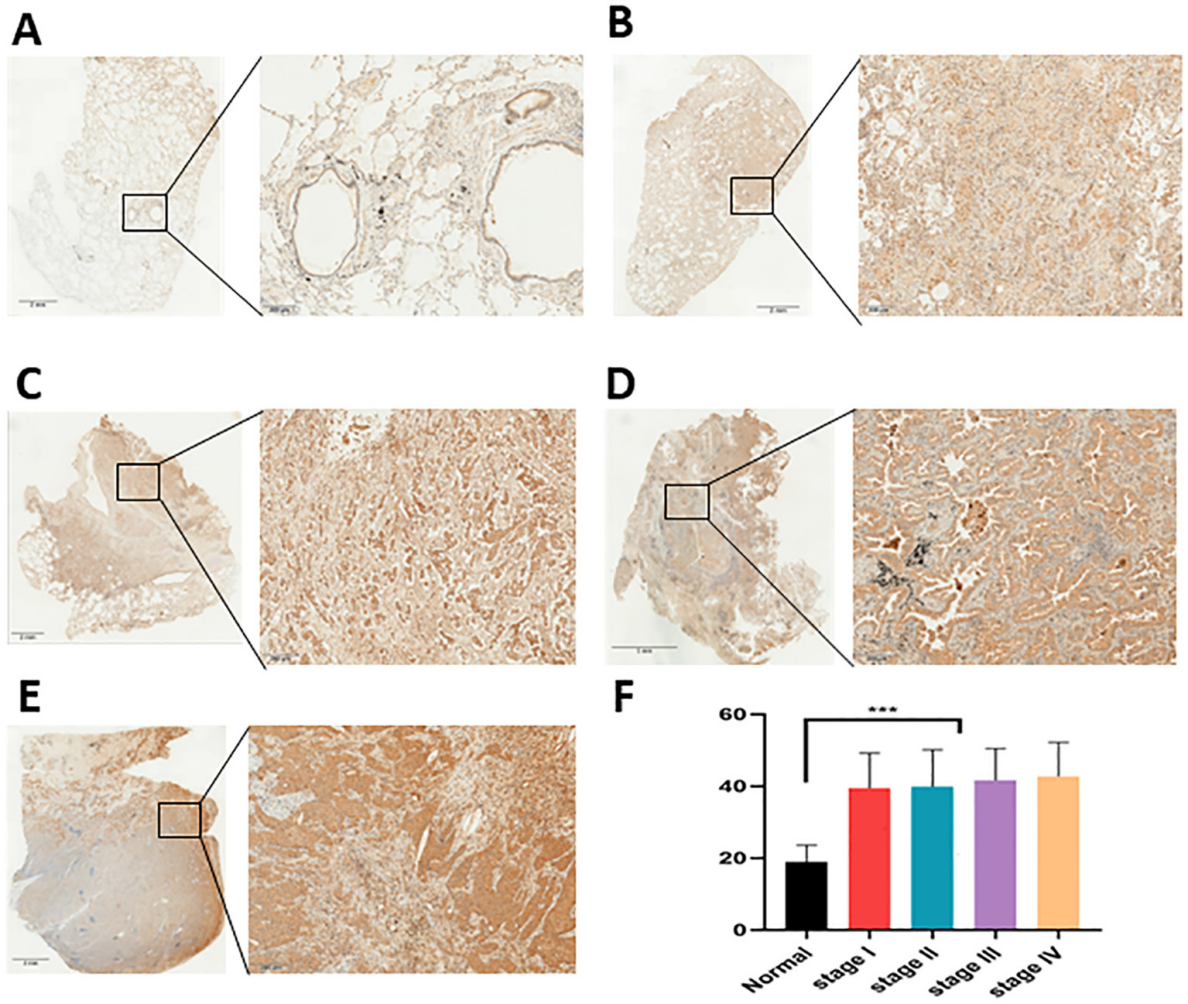
TNS4 expression analysis via Immunohistochemistry. **(A−E)** Comparative expression of TNS4 in lung adenocarcinoma (LUAD) tissues versus normal lung tissues. **(F)** Statistical analysis results showing differences in TNS4 expression between normal lung tissue and lung cancer across various stages (***p<0.001).

**Figure 7:**
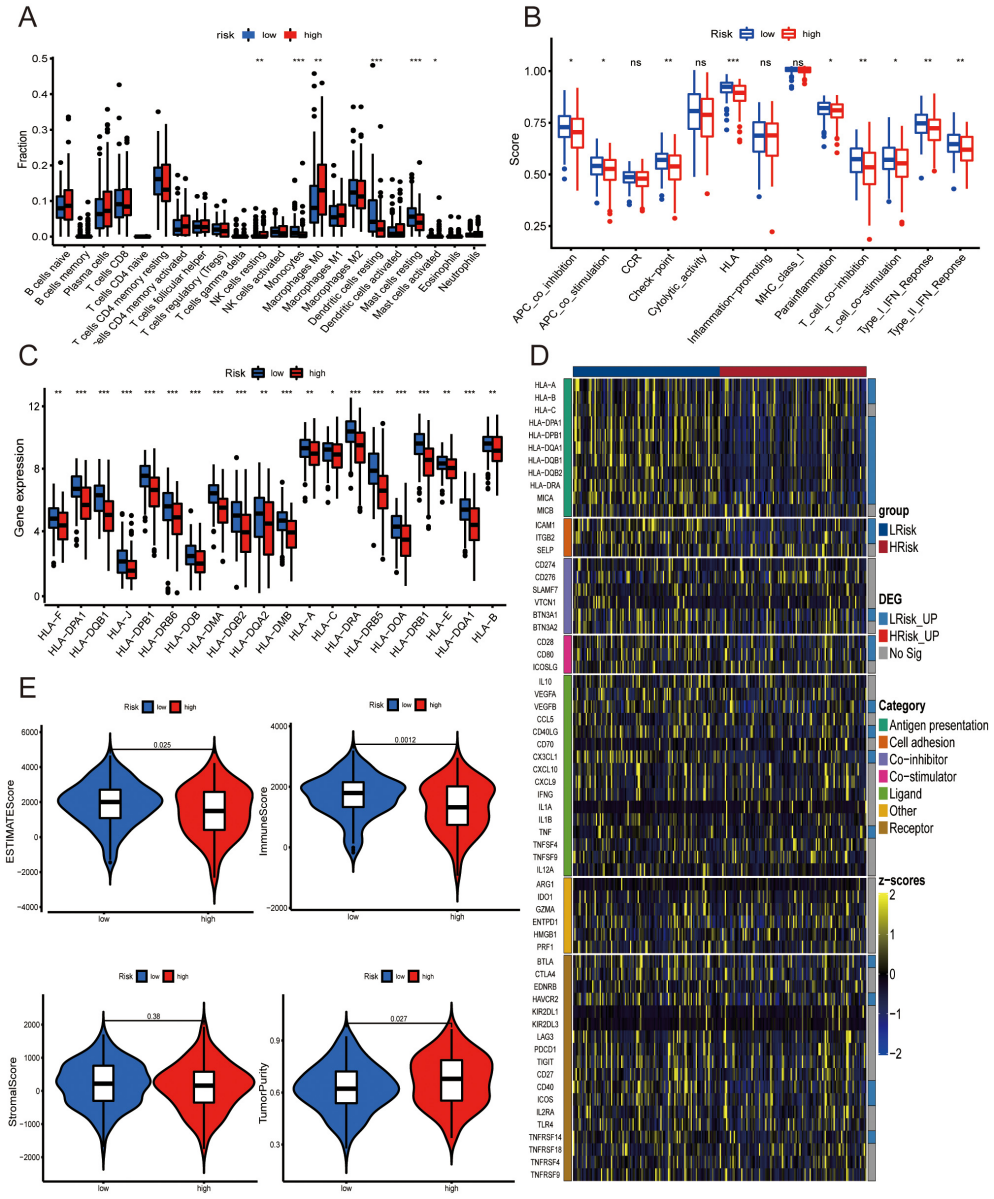
Relationship between risk score and tumor immune microenvironment. **(A)** Box plot displaying the estimated proportions of 22 immune cell types within high- and low-risk groups. **(B)** Box plot showing differences in immune-related ssGSEA scores between groups. **(C)** Box plot illustrating variations in HLA-related gene expression levels between groups. **(D)** Heatmap depicting disparities in immune checkpoint expression across the groups. **(E)** Violin plot highlighting differences in ESTIMATE score, immune score, stromal score, and tumor purity between the high- and low-risk groups (ns, no significant difference; *p<0.05; **p<0.01; ***p<0.001).

**Figure 8:**
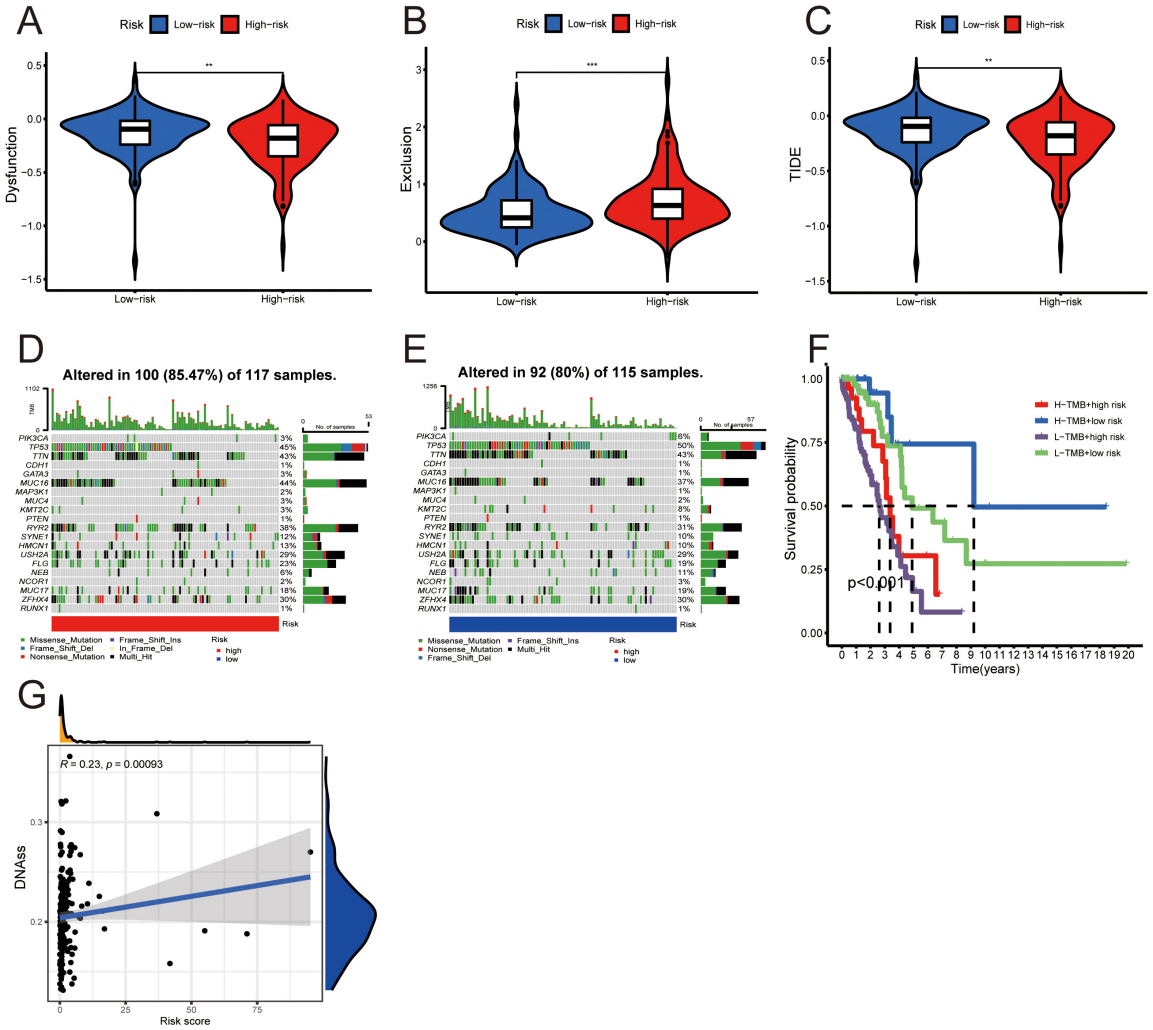
Risk scores, tumor microenvironment, and genetic mutations. **(A−C)** Violin plots illustrating differences in dysfunction, exclusion, and TIDE metrics between the high- and low-risk groups (Wilcoxon test, ^**^ *p* < 0.01, ^***^ *p* < 0.001). **(D, E)** Waterfall charts depicting mutation frequencies in high- and low-risk groups. **(F)** Kaplan-Meier survival curve assessing the combined effect of tumor mutation burden (TMB) and risk score on patient prognosis (log-rank test, *p* < 0.05). **(G)** Scatter plot correlating risk score with DNA methylation-based stemness score (DNAss).

### 3.6 Genomic mutation analysis

Somatic mutation data revealed differing mutation frequencies between the high- and low-risk LUAD groups, displayed through waterfall charts. In the high-risk group, 85.47% (100 out of 117) of samples showed mutations, with missense mutations being the most prevalent (**Figures 8D**). TP53 mutations were notably high at 45%, following TTN at 43%. Conversely, the low-risk group had an 80.00% (92 out of 115) mutation rate, with TP53 mutations most frequent at 50% (**Figures 8E**). Survival analysis demonstrated that low-risk patients with high TMB had significantly better outcomes than those in the high-risk group with low TMB (*p* < 0.001, **Figure 8F**). Additionally, our analysis showed a positive correlation between risk score and DNA methylation-based stemness score (DNAss) in LUAD (**Figure 8G**).

### 3.7 Prognostic significance of TNS4 in LUAD

**Figure 9:**
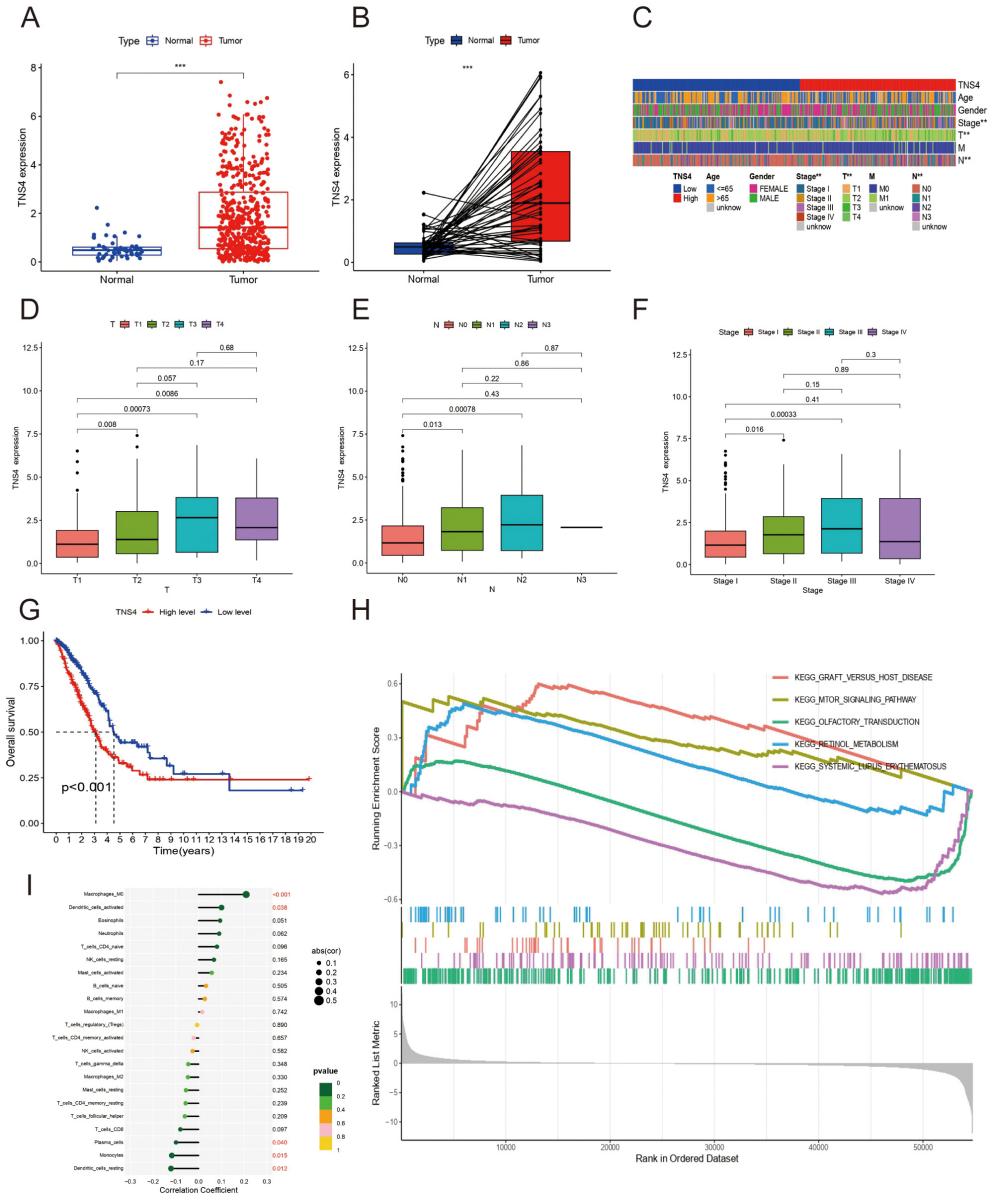
TNS4 expression and clinical implications in Lung Adenocarcinoma (LUAD). **(A, B)** Box plots demonstrating significantly elevated TNS4 expression in tumor tissues compared to normal tissues. **(C−F)** Association of higher TNS4 expression with advanced clinicopathological features in LUAD. **(G)** Kaplan-Meier plots linking elevated TNS4 levels with poorer prognostic outcomes. **(H)** Gene set enrichment analysis. **(I)** Bar graph displaying correlations between TNS4 expression levels and immune cell infiltration.

TNS4 mRNA levels were significantly higher in LUAD tissues compared to normal lung tissues (**Figures 9A, B**). This expression correlated significantly with pathological stages of the disease (**Figures 9C–F**). Immunohistochemical staining conducted at Jiangmen Central Hospital confirmed higher TNS4 protein levels in LUAD than in normal lung tissue (**Figures 6A, F**), though there was no statistical difference in expression across different tumor stages (**Figures 6A–F**). Kaplan-Meier survival curves indicated that lower TNS4 expression was linked to improved prognosis (**Figure 9G**). GSEA identified significant enrichment of graft-versus-host disease and mTOR signaling pathways in the high TNS4 expression group, while pathways like olfactory transduction and retinol metabolism were prominent in the low expression group (**Figure 9H**). Immune infiltration analysis demonstrated positive correlations of TNS4 expression with M0 macrophages and activated dendritic cells, and negative correlations with plasma cells, monocytes, and resting dendritic cells. These findings collectively suggest that TNS4 not only serves as a marker of tumor progression in LUAD but also influences the tumor microenvironment, potentially impacting patient response to immunotherapy and overall survival.

### 3.8 Functional implications of TNS4 knockdown in LUAD

**Figure 10:**
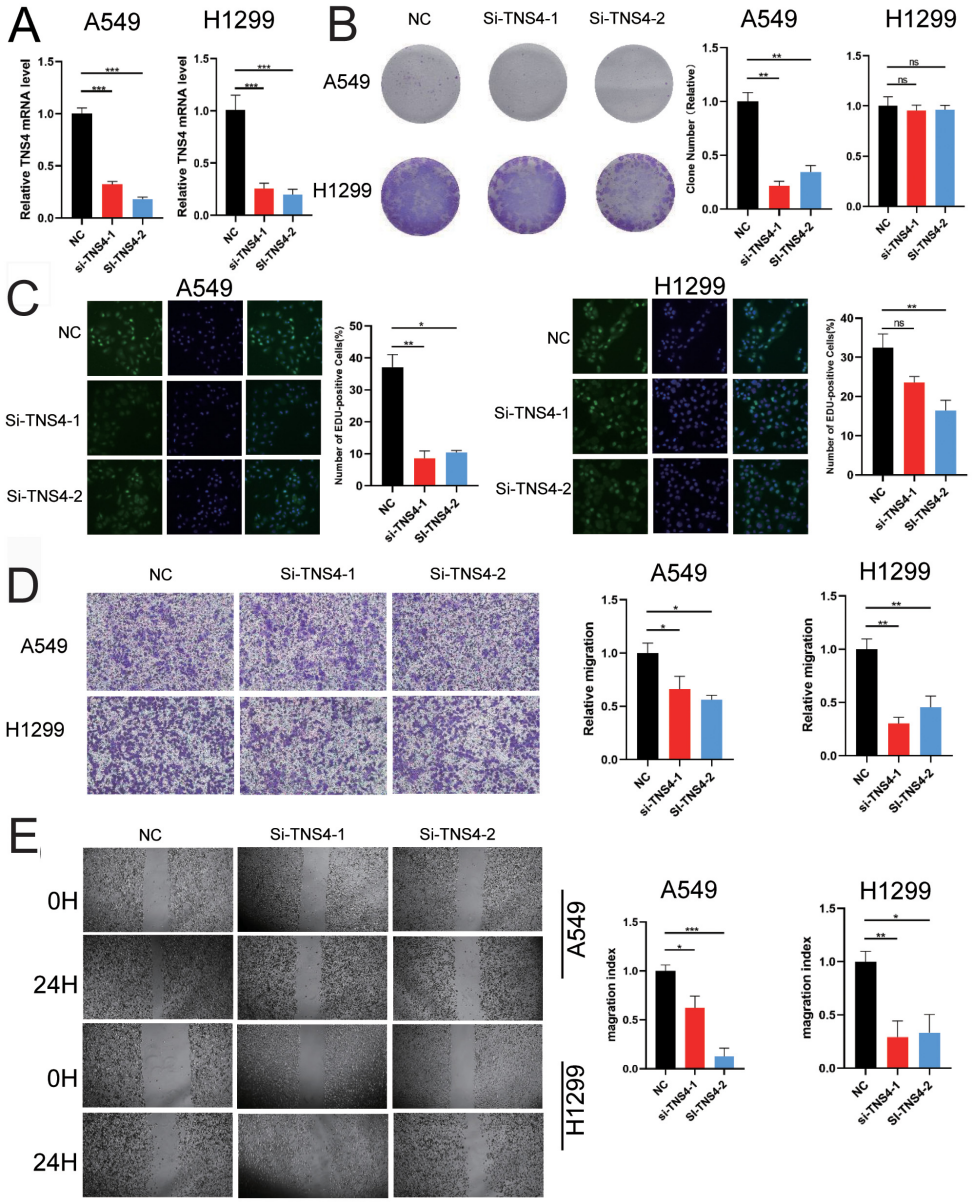
Impact of TNS4 knockdown on Lung Adenocarcinoma (LUAD) cell proliferation and migration. **(A)** RT-qPCR results confirming the efficiency of TNS4 knockdown in A549 and H1299 cells using two siRNAs. **(B)** Colony formation assays in A549 and H1299 cells, analyzed with ImageJ. **(C)** EdU assay measuring cell proliferation in control versus TNS4 knockdown cells. **(D)** Transwell assay comparing cell migration in control and TNS4 knockdown cells. **(E)** Wound healing assay illustrating migration differences between control and TNS4 knockdown cells (ns, no significant difference; *p<0.05; **p<0.01; ***p<0.001).

To investigate TNS4’s role in LUAD, we knocked down its expression using two specific siRNAs in A549 and H1299 cell lines. RT-qPCR verified the effective reduction of TNS4 levels (**Figure 10A**). This knockdown resulted in decreased cell proliferation and colony formation in A549 cells (**Figure 10B**). EdU assays further validated the reduction in proliferation post-TNS4 knockdown (**Figure 10C**). Moreover, transwell and wound-healing assays showed that TNS4 silencing reduced migratory capabilities in both A549 and H1299 cells (**Figures 10D, E**). These results highlight TNS4’s vital role in promoting cell proliferation and migration in LUAD, suggesting its potential as a therapeutic target for treating the disease.

## 4 Discussion

LUAD is the predominant form of lung cancer, notorious for its aggressive behavior and generally poor outcomes (36). Due to its asymptomatic early stages, LUAD is frequently detected only in more advanced phases, complicating early diagnosis and prognosis (37). Disulfidptosis, a newly identified type of cell death, has been recognized as a potential critical determinant in this area (38–40). Our research explores disulfidptosis in LUAD, evaluating its capability to forecast disease prognosis, patient survival, and the efficacy of immunotherapy treatments. Understanding disulfidptosis may unlock new avenues for precise interventions, offering valuable insights for the development of both diagnostic tools and therapeutic approaches in treating LUAD.

In our study, the proposed prognostic model was validated using Kaplan-Meier survival curves, showing significantly lower OS rates in the high-risk group compared to the low-risk group (*p* < 0.001). Validation of the model’s robustness was further confirmed using a validation set. Subgroup analysis revealed the model’s excellent predictive value across various categories, including age groups (>65, ≤65), gender (male, female), and cancer stages (N0, M0, I-II, T1-2) (p < 0.05). The analysis of immune infiltration identified significant differences in immune cell profiles between the high- and low-risk groups.

Additional studies focused on TNS4, the central gene in our model, identified as a multifunctional cytokine involved in enhancing tumor invasion and metastasis in several cancers, including head and neck squamous cell carcinoma, gastric, pancreatic, and colorectal cancers, where it promotes tumor invasion and metastasis (41–44). TNS4 functions through complex mechanisms involving multiple pathways and cytokines that regulate its expression. It plays a critical role in epithelial-mesenchymal transition (EMT) in tumors, influencing pathways such as FAK activation which, in turn, enhances PI3K/Akt and TGFβ signaling, promoting tumorigenesis (45). TNS4 also upregulates Src expression to facilitate colorectal cancer metastasis and depends on Ras/MAPK signaling for its activity (46). In gastric cancer, TNS4 contributes to disease progression by upregulating p-AKT, p-GSK-3β, and β-catenin (47). Additionally, in esophageal cancer, it activates the EGFR-EFNA1/EPHA2-VEGFA signaling pathway, enhancing tumor cell proliferation, migration, and invasion (48). However, studies on TNS4’ relationship with LUAD are limited. Our results indicate that elevated TNS4 expression in LUAD patients correlates with poorer prognosis and clinical characteristics. Immunohistochemical analysis in our study showed TNS4 levels are higher in LUAD tissues than in normal lung tissues, consistent with other related studies (49). Importantly, TNS4 expression in LUAD did not vary significantly across different tumor stages, indicating that its high expression in tumor tissues is not stage-dependent. Moreover, we conducted experiments on A549 and H1299 cell lines to assess cell proliferation and invasiveness. Colony Formation and EdU assays revealed that TNS4 interference significantly curtails the proliferative capabilities of both A549 cells. Furthermore, wound healing and transwell assays showed that suppression of TNS4 greatly diminishes the migratory and invasive abilities of these cells. TNS4, a disulfidptosis-related gene, is overexpressed in LUAD tissues and promotes LUAD cell proliferation and invasiveness. However, the specific mechanisms by which TNS4 induces disulfidptosis warrant further experimental exploration. Additionally, while TNS4 shows promise, its effectiveness as a standalone marker in clinical settings has yet to be fully validated. To establish its predictive power and reliability, comprehensive validation through larger, multicenter studies is essential. Considering the complex mechanisms of tumor development and the multifaceted nature of malignancy, TNS4 emerges as a potential predictive marker for LUAD, though its role in disease progression requires further comprehensive assessments. The integration of disulfidoptosis-related biomarkers like TNS4 into existing diagnostic and therapeutic protocols represents a promising avenue to enhance the precision of lung cancer management. This necessitates a systematic approach to evaluate these biomarkers across various clinical scenarios to fully understand their potential in predicting treatment responses and patient outcomes.

## 5 Conclusion

This research delineates the classification of two molecular subtypes based on genes regulated by disulfidptosis and conducts functional enrichment analysis. Additionally, a risk model was developed to forecast the prognosis and therapeutic outcomes for patients with LUAD, achieving high accuracy and robustness upon validation. Immunohistochemistry and *in vitro* experiments confirmed the central gene TNS4 as a potential therapeutic target. These results indicate that genes regulated by disulfidptosis could serve as promising biomarkers and therapeutic targets for LUAD.

## Data Availability

The original contributions presented in the study are included in the article/supplementary material. Further inquiries can be directed to the corresponding authors.
